# Measuring the Response of Extrahepatic Symptoms and Quality of Life to Antiviral Treatment in Patients with Hepatitis C

**DOI:** 10.1155/2013/910519

**Published:** 2013-10-07

**Authors:** David Isaacs, Nader Abdelaziz, Majella Keller, Jeremy Tibble, Inam Haq

**Affiliations:** ^1^Brighton and Sussex Medical School, Brighton BN1 9PX, UK; ^2^Medicine, Royal Sussex County Hospital, Brighton BN2 5BE, UK

## Abstract

*Background*. HCV infection is associated with musculoskeletal manifestations such as chronic widespread pain, sicca syndrome, polyarthritis, and a reduced HRQOL. Little data is available on the effect of treatment on these manifestations. This study measured changes in extrahepatic symptoms and HRQOL before and after antiviral treatment in a large UK patient cohort. *Methods*. 118 patients completed HQLQ and rheumatological questionnaires before and after treatment with pegylated interferon-**α** and ribavirin, with specific regard to chronic widespread pain, sicca syndrome, and sustained virological response. *Results*. There was significant improvement in HQLQ domains of physical functioning, physical disability, social functioning, limitations and health distress due to hepatitis, and general health. There was significant deterioration in domains of positive well-being, health distress, and mental health. There was a significant decline prevalence of CWP (26.3% versus 15.3%, *P* = 0.015). Sicca syndrome prevalence fell insignificantly (12.7% versus 11%). SVR was associated positively with all HRQOL changes and significantly with CWP remission. *Conclusions*. HCV antivirals significantly improve poor HRQOL scores and CWP. Before treatment, both were common, coassociated, and unaccounted for through mixed cryoglobulinemia alone. Although a role of the hepatitis C virus in CWP cannot be deduced by these results, symptomatic improvement via antiviral treatment exists for this subset of patients.

## 1. Introduction

Past clinical understanding confined the burden of chronic hepatitis C infection (HCV) to later stages of hepatic impairment. Now it is known that HCV's extrahepatic manifestations (EHMs) and reduced health-related quality of life (HRQOL) often develop before hepatic impairment [1]. Diagnostically, this understanding helps uncover underlying HCV in people presenting with associated EHMs and vice versa. Prognostically, it is unclear whether EHMs independently lower HRQOL in HCV patients and whether they respond to antiviral therapy with or without a sustained virological response (SVR). Answers to these questions would help to determine whether the burden of EHMs in HCV patients merits additional management approaches beyond antiviral therapy. 

HRQOL reduction in HCV is multifactorial. Poor baseline HRQOL is partly psychosocial in origin, relating to the stigma of illness, a history of illicit drug use for a large proportion of patients, and high rates of fatigue, anxiety, and depression [2, 3]. Mood-related aspects of HRQOL may even be organically mediated by HCV colonization of brain microglia and activation of brain interleukins [4]. However, HRQOL is also impaired through somatic symptoms, which may be specific to HCV pathophysiology. For example, HCV patients score worse than hepatitis B patients on somatic symptoms of the SF-36 questionnaire, and patients unaware of their HCV infection still score worse than the general population [2, 5]. Furthermore, most evidence points to an improvement in HRQOL as being associated with SVR after treatment with pegylated interferon alone or with ribavirin [2, 6–9], which was confirmed by a meta-analysis that also suggests that a minimum change of 4.2 points on the SF-36 vitality scale is needed for a significant improvement in HRQOL [3]. Several trials, however, show improvements in HRQOL independently of SVR, raising the hypothesis that viral suppression alone can achieve significant physiological changes [6, 8, 10, 11].

HCV associated EHMs create somatic symptoms that probably contribute to patients' lower HRQOL scores, although this has not been sufficiently assessed. Arthralgia and myalgia are among the common rheumatological symptoms associated with HCV, with one study displaying a prevalence of 23% and 15% for each, respectively [12]. Less common rheumatological EHMs include arthritis, vasculitis, sicca syndrome, Sjögren's syndrome, and systemic lupus erythematosus [12, 13]. 

Mixed cryoglobulinaemia (MC), which has been found in 1–60% of people with HCV, describes the presence of IgM immunoglobulins (rarely IgG or IgA) that form complexes with monoclonal (type II) or polyclonal (type III) rheumatoid factors (RFs) [12]. Types II and III MC are far commoner than simple cryoglobulinaemia (type I) that does not include RF complexes and is not associated with HCV. However, reported prevalence of MC depends heavily on the accuracy of laboratory techniques in cryoprecipitation at low temperatures and measuring cryoglobulin concentrations. 

Deposition of MC immune complexes, alongside poor clearance and reduced complement fragments, can result in small-vessel vasculitis and study populations with idiopathic MC have high reported incidences for cutaneous, musculoskeletal, and renal manifestations. Therefore, secondary MC may play a common role in EHMs as 5–10% of HCV patients display an overt MC vasculitic triad of weakness, arthralgia, and purpura [13, 14]. Multiple tropism of the virus, particularly to lymphocytes, may account for many EHMs with or without development of MC [15]. The HCV virus seems to facilitate a state of increased autoantibody and cryoglobulin production by expanding B cells through the envelope protein E2 interacting with the CD81 receptor and increasing B-cell survival by activating the Bcl2 proto-oncogene [13, 16, 17]. A similar state may also be achieved by the virus molecular mimicry of host autoantigens [17, 18]. As of yet, no factors specific to host background, environment, or viral genotype have been associated with the emergence of EHMs [17].

Chronic pain in particular has been shown to impair HRQOL in HCV patients [19]. However, apart from a specific role for MC in some cases of arthralgia, there is no overarching evidence for a pathogenic role of the virus in most presentations of chronic pain. For example, histopathological presence of HCV in muscles of myalgia sufferers [13, 20] and an increased prevalence of HCV in fibromyalgia patients have not been consistently found [21, 22].

There have been limited studies on the role of antiviral therapy for EHMs. Antiviral therapy has some evidence for improving pain in the context of MC complicated HCV. In one study, arthralgia and myalgia improved for approximately half the patients, and in those achieving SVR fatigue and MC levels dropped significantly [23]. In another study, interferon and ribavirin therapy cleared MC in 37.8% and arthralgia in 80% of patients [24]. However, to our knowledge no other studies have yet assessed therapeutic effects on EHMs alongside measured HRQOL changes in HCV patients. 

## 2. Methods

### 2.1. Aims

1. Investigate whether HRQOL improves following antiviral therapy.
2. Investigate whether extrahepatic outcomes improve following antiviral therapy.
3. Determine whether an association exists between extrahepatic symptoms and HRQOL before and after treatment.
4. Investigate whether improvement in HRQOL or extrahepatic symptoms is dependent on SVR after treatment.

### 2.2. Ethics

A study protocol for a cross-sectional epidemiological study into the prevalence of musculoskeletal symptoms among HCV positive adults in a Brighton cohort was proposed and granted ethics approval by the Brighton East Research Ethics Committee in January 2006 (reference. 06/Q/1907/134).

Out of a cohort of 537 HCV patients managed at the Digestive Diseases Unit at the Brighton and Sussex University Hospital Trust (BSUH), in the UK, we assessed the results of 118 patients who were not coinfected with HIV or HBV and who had completed standard antiviral treatment with pegylated interferon-*α* and ribavirin.

### 2.3. Outcomes

Participants answered the hepatitis quality of life questionnaire (HQLQ) [25] and a survey of symptoms affecting the spine, muscles, bones, and joints before treatment and six months after finishing treatment. The HQLQ is generically based on SF-36 health survey but is validated as a hepatitis-specific instrument in the measurement of HRQOL [8]. Outcome measures included presence of chronic widespread pain (CWP) according to the Manchester criteria (pain in the axial skeleton and at least two contralateral body quadrants for at least 3 months), the number of affected joints, pain intensity, and interference with daily life as scored on a visual analogue scale (VAS). The binary CWP Manchester criteria outcome was preferred over assessments of fibromyalgia, as the latter would require validation via physician led examinations (self-reports of having a diagnosis of fibromyalgia at baseline were not considered). A lack of physical examinations also excluded the presence of vasculitic rashes from our assessments. In the context of this study, patients were considered positive for sicca syndrome if they reported both mouth and eye sicca symptoms using standardized questions into xeropthalmia and xerostomia; a full assessment of Sjögren's with antiRO/SSA or other autoantibodies was not performed. 

### 2.4. Statistics

A paired samples *t*-test or Wilcoxon test was used to analyze changes in HRQOL, VAS, and number of painful joints from before treatment to 6 months after treatment. An independent samples *t*-test or Mann-Whitney *U* test (nonparametric) was used to analyze the relationship between HRQOL changes and SVR. A Pearson chi-square test was used in the number of patients with CWP, number of patients with pain for more than 3 months, or number of patients who agreed with the statement “I ache all over.” Exact *P* values of <0.05 in the two-tailed tests were considered significant. 

## 3. Results

### 3.1. Background Information

See Table 1. Our cohort reflected a relatively young population with a mean age of 46 and an overwhelming proportion with a history of intravenous drug use—due to Brighton having one of the highest prevalence of IVDU in the UK [26]. Unemployment was also prevalent at 41%. Furthermore, 18% of patients had preexisting arthritis. Although the presence of RF was common (see Table 2), MC prevalence was low at only 5.9%, though laboratory techniques and unsatisfactory test completion rates should be taken into account. 

### 3.2. HRQOL Outcomes

**Figure 1:**
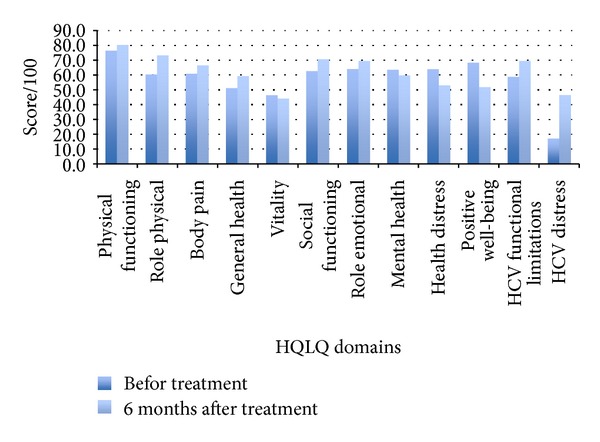
HQLQ scores before and after treatment.

There was a statistically significant improvement in scores in the following 6 out of the 12 domains of the HQLQ: physical functioning, physical disability, social functioning, limitations due to hepatitis, health distress due to hepatitis, and general health (see Table 3 and Figure 1). There was a statistically significant deterioration in 3 of the domains (positive well-being, health distress, and mental health), and there was no significant change in the rest of the domains (body pain, role emotional, and vitality). 

### 3.3. Rheumatological Outcomes

There were a high baseline prevalence of chronic pain symptoms and statistically significant declines after treatment in the number of patients with CWP (11% reduction, *P* = 0.015), number of patients with pain for more than 3 months (11% reduction, *P* = 0.041), or number of patients who agreed with the statement “I ache all over” (10.1%, *P* = 0.029) (see Table 4). 

Having CWP before treatment was significantly associated with worse pretreatment pain levels (5.7/10 versus 2.7/10, *P* > 0.001) and their interference with daily life as reported on VAS (5.0/10 versus 2.1/10, *P* > 0.001), though the overall VAS pain scores and the average number of painful joints did not fall significantly after treatment. Having CWP before treatment was also negatively associated with all HQLQ domains (significantly in all except vitality and health distress and hepatitis-specific distress), except for positive well-being where people with CWP scored significantly higher (75/100 versus 66/100, *P* = 0.023) (see Table 5). 

**Figure 2:**
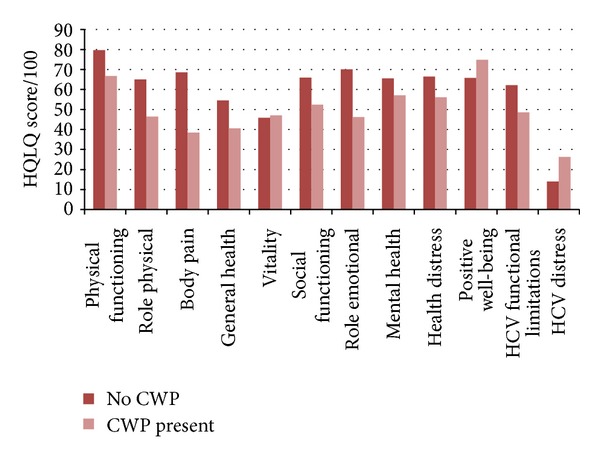
Pretreatment HQLQ scores in people with CWP versus no CWP.

A remission of CWP after treatment was significantly associated with improvements in the HQLQ domain of body pain (15.4 improvement in CWP remission versus 11.8 if it stays the same and 14.8 reduction if CWP develops, *P* = 0.024), physical function (*P* = 0.043), and nearly role emotional (*P* = 0.052) (see Figure 2). In contrast to pretreatment CWP, having CWP after treatment was only significantly associated with a worse role physical and body pain HQLQ score (*P* = 0.008, *P* < 0.001).

There were a small number of patients who matched the study's criteria for sicca syndrome, and the decline after treatment was not significant (12.7% versus 11%, *P* = 0.804). 

### 3.4. Associations with SVR

**Figure 3:**
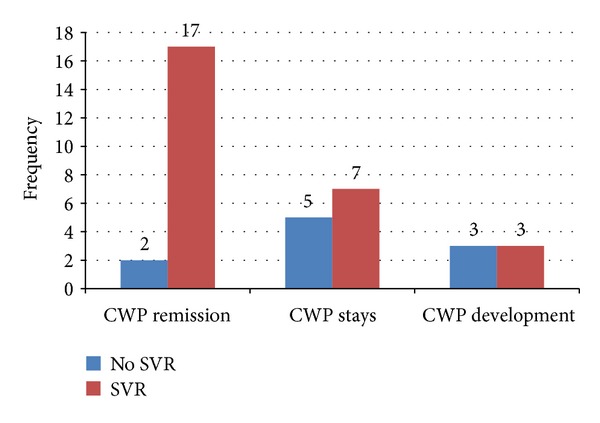
CWP progression versus SVR.

After treatment 67% of patients achieved SVR. There were positive nonsignificant associations between SVR and changes in all HQLQ scores and significant positive associations between SVR and changes in CWP (*P* = 0.038) (see Figure 3). However, achieving SVR was not associated with the minor regression in sicca symptoms.

## 4. Discussion

### 4.1. Quality of Life

Patients in this study showed significant improvement in 6 of the 12 domains of HQLQ following treatment, which on the whole improved the total score. However, these improvements did not include an increase of more than 4.2 points on the vitality scale, which has been defined by Spiegel et al. as the minimally clinically important difference in HRQOL [3]. Therefore, to argue that in this case antiviral therapy has had a positive effect on HRQOL is dependent on the weighting given to different domains. 

In keeping with previous studies, HRQOL improvement was seen in the domains relating to physical health [2, 6, 7]. Given the significant improvement in domains relating to general health, disease limitations, social functioning, and hepatitis-related distress, the deterioration in domains relating to mental well-being and positive well-being distress suggests a complex range of effects with antiviral treatment. This may be an exacerbation of a high baseline incidence of anxiety and depressive symptoms, which are commonly reported among HCV-infected patients [11, 27]. These baseline symptoms may be related to a patient's distress at being diagnosed with a chronic and serious illness [28], a history of illicit drug use [29], or a direct effect of the virus [30]. The exacerbation of these symptoms, on the other hand, may be caused by antiviral treatment itself, as interferon alpha is known to cause depression [27]. Our results support the hypothesis that initial impairments in physical domains are more likely to be virus related than impairments in domains of mental health given that the former domains improved following treatment while the latter did not [1, 31]. 

### 4.2. Rheumatological Symptoms

Our results reflect the direction of previous findings that fewer patients experience myalgia and arthralgia following treatment [23, 24, 32]. Interestingly, however, while patients with CWP, 3-month pain and “I ache all over” reported that pain levels all fell significantly, the average VAS pain intensity and impact levels, bodily pain aspects of the HQLQ, and the number of painful joints were all low and changed little with treatment. This demonstrates that extrahepatic pain manifestations in HCV patients pool together in a subset of patients and are unaltered by treatment in the majority of cases. 

An analysis comparing patients with and without CWP before treatment revealed baseline HQLQ scores to be significantly worse in 9 domains in those with CWP, though whether this was cause or effect is not ascertainable here. The discrepancy of a greater positive well-being in patients with baseline CWP represents a psychological anomaly as mental health and other psychological domains were significantly worse in this subset. CWP remission after treatment was also significantly associated with an improved body pain and physical function score, which changed little otherwise for the whole cohort. This shows that having CWP before treatment is significantly associated with a worse HRQOL and that the bodily pain aspect may be particularly responsive to treatment in this subset, which has not been illustrated in the literature before. The clinical implications for this lie in better recognition of this subset of patients in order to discuss the potential effects of treatment for them specifically and in using appropriate additional management strategies such as analgesia or exercise. 

If the observed trends in pain reflect the effects of a viral immunomodulatory process affecting a subset of patients, one would expect more patients to also undergo remission of sicca syndrome. A lack of response to treatment may be due to unknown factors and an already underwhelming sicca prevalence in the cohort relative to other studies. [33]. However, a more evident response would be expected because sicca syndrome has an established viral pathogenesis where HCV exocrine gland tropism is strongly associated with lymphocytic infiltration, sometimes in combination with rheumatoid factor and autoantibodies and resultant xerostomia and xerophthalmia [34]. Arthralgia on the other hand is more known to be associated with HCV through a mechanism of elevated autoantibodies and MC that promotes tissue injury, a biomarker which was rare in our cohort [35]. Thus, our observed improvements in pain scores may also be due to unmeasured nonvirological changes, such as nutritional status improvements, reduced alcoholic consumption, and subsequent improvements in vitamin D levels that are associated with nonspecific musculoskeletal pain [36].

### 4.3. SVR Discussion

Data from a meta-analysis of HRQOL studies in HCV patients has suggested that SVR is associated with improved HRQOL. In this study, there was a general trend in that direction without statistical significance, which reflects the power of the sample size. Our results suggest that antiviral therapy can improve HRQOL and pain scores in the absence of SVR, which may be due to a placebo response, which this study did not control for, or due to the immunomodulatory effects of antiviral therapy, which have not been fully described yet. For example, interferon has been found to have antiproliferative effects on MC and has been used on MC even before link with HCV was found. Furthermore, it has been suggested that this effectiveness may be irrespective of interferon's antiviral properties [37]. Conversely, other studies have shown that MC and subsequent vasculitic symptoms can persist despite SVR [38], which supports the hypothesis of there being both memory (virus-dependent) and naïve (virus-independent) autoimmune B cell expansion after HCV infection [24].

## 5. Limitations

The main drawback of this study is the lack of a matched control group not undergoing antiviral treatment, due to there being only a minority of such patients, of whom many have concurrent psychiatric illness, IVDU, and alcoholism. Furthermore, concomitant use of vitamin D supplementation, analgesia, and anti-inflammatories during questionnaire completion was not controlled for. Due to the small proportion of patients with the extrahepatic symptoms in this study, a larger sample may be necessary to improve the power of measuring the effects of treatment and SVR. Therefore, larger study participation with controls and longer followup are needed to validate the study's results.

## 6. Conclusions

Antiviral therapy with pegylated interferon-*α* and ribavirin can significantly improve physical and functional aspects of HRQOL as well as rheumatological CWP symptoms in HCV patients, despite no changes in vitality and deterioration in mental health and positive well-being. In our cohort, CWP prevalence is high, demonstrably related to HRQOL, and unaccounted for through cryoglobulinaemia alone. A role for the virus in CWP is purely speculative on the basis of these results, and other potential causes such as vitamin D deficiency were not measured. Of relevance to clinical practice, however, are the study's observed CWP prevalence and response in HCV patients undergoing antiviral treatment. An awareness of this finding may prompt earlier diagnosis and investigation of CWP in HCV patients and warrant chronic pain relief as an additional indication for antiviral treatment besides existing benefits on hepatitis and HRQOL. Additional management strategies such as graded exercise and analgesia should be discussed in this subset of chronic pain sufferers for whom antivirals are deemed inappropriate. 

## Figures and Tables

**Table 1:** Background information.

Background variable	% (*n*)
Age	
Mean = 46	
Gender	
Male	59 (69)
Female	41 (48)
Ethnicity	
White British	79 (90)
White Irish	3 (4)
White other	12 (14)
Other	6 (6)
In employment	59 (68)
Transmission mode	
Intravenous drugs	77.8 (49)
Blood transfusion	3.2 (2)
Homosexual sex	6.3 (4)
Heterosexual sex	1.6 (1)
Other	8 (7)
Missing	(55)
Genotype	
1	36.5 (19)
2	3.8 (2)
3	26.9 (14)
2, 3	30.8 (16)
4	1.9 (1)
Missing	(66)
SVR	
Achieved	67 (76)
Not achieved	33 (38)
No record	(4)
Arthritis	
Osteoarthritis	5 (6)
Rheumatoid	5 (6)
Unknown	5 (6)
Other	2.5 (3)
Positive for cryoglobulins	5.9 (7)
Inflammatory bowel disease	5.9 (7)

**Table 2:** Background serology.

Assay	Abnormal result *N* (total found)	Normal reference range	Value taken as abnormal result
Rheumatoid factor	48 (74)	<15 IU/mL	>14 IU/mL
ANA	5 (76)	Nil	>1 : 160
ENA	4 (65)	Nil	+
Mixed cryoglobulinemia	7 (82)	Nil	+
C3 low	4 (81)	0.75–1.65 g/L	<0.75 g/L
C4 low	14 (80)	0.14–0.54 g/L	<0.14 g/L

**Table 3:** HQLQ scores before and after treatment.

HQLQ domain	Mean score		Significance of change
	Before treatment	6 months after treatment	*P* value
Physical functioning	76.3	80.2	0.024
Role physical	60.2	73.1	0.002
Body pain	60.6	66.3	0.055
General health	50.9	59.1	>0.001
Vitality	46.1	43.9	0.233
Social functioning	62.4	70.4	0.009
Role emotional	63.8	69.3	0.350
Mental health	63.3	59.5	0.048
Health distress	63.8	52.8	0.008
Positive well-being	68.1	51.6	>0.001
Hepatitis-specific functional limitations	58.6	69.3	>0.001
Hepatitis-specific distress	17.2	46.3	>0.001

**Table 4:** EHMs before and after treatment.

Extrahepatic symptom	Prevalence/mean score		Significance of change (*P* value)
	Before treatment	6 months after treatment	
CWP	26.3%	15.3%	0.015
Sicca syndrome	12.7%	11%	0.804
Average pain intensity in past month (0–10)	3.5	3.03	0.135
Interference with daily activities in past month (0–10)	2.91	2.67	0.48
Number of painful joints in past month	2.59	2.24	0.306
Pain for more than 3 months	64.4%	53.4%	0.041
“I ache all over”	23.7%	13.6%	0.029

**Table 5:** Variables associated with pretreatment CWP.

	CWP before treatment	No CWP before treatment	Statistical significance (*P* value)
No. of painful joints	5.19	1.67	>0.001
VAS pain rating (mean)	5.7/10	2.7/10	>0.001
VAS interference rating (mean)	5.0/10	2.1/10	>0.001
HQLQ domain			
Physical functioning	66.8	79.7	0.025
Role physical	46.5	65.1	0.025
Body pain	38.5	68.6	>0.001
General health	40.6	54.6	0.002
Vitality	47.1	45.8	NS (0.553)
Social functioning	52.4	65.9	0.028
Role emotional	46.2	70.1	0.005
Mental health	57.2	65.5	0.035
Health distress	56.1	66.5	NS (0.182)
Positive well-being	74.8	65.7	0.023
Hepatitis-specific functional limitations	48.5	62.2	0.014
Hepatitis-specific distress	26.3	14.0	NS (0.064)

## Abbreviations

HCV: Hepatitis C virus
EHMs: Extrahepatic manifestations
CWP: Chronic widespread pain
HRQOL: Health-related quality of life
HQLQ: Hepatitis quality of life questionnaire
SVR: Sustained virological response
MC: Mixed cryoglobulinaemia
SS: Sicca syndrome.
